# Immuno-antibiotics: targeting microbial metabolic pathways sensed by unconventional T cells

**DOI:** 10.1093/immadv/ltab005

**Published:** 2021-04-05

**Authors:** Matthias Eberl, Eric Oldfield, Thomas Herrmann

**Affiliations:** 1 Division of Infection and Immunity, School of Medicine, Cardiff University, Cardiff, UK; 2 Systems Immunity Research Institute, Cardiff University, Cardiff, UK; 3 Department of Chemistry, University of Illinois at Urbana-Champaign, Urbana, IL, USA; 4 Institut für Virologie und Immunbiologie, Julius-Maximilians-Universität Würzburg, Würzburg, Germany

**Keywords:** γδ T cells, MAIT cells, microbial infection, antibiotics, immunotherapy

## Abstract

Human Vγ9/Vδ2 T cells, mucosal-associated invariant T (MAIT) cells, and other unconventional T cells are specialised in detecting microbial metabolic pathway intermediates that are absent in humans. The recognition by such semi-invariant innate-like T cells of compounds like (*E*)-4-hydroxy-3-methyl-but-2-enyl pyrophosphate (HMB-PP), the penultimate metabolite in the MEP isoprenoid biosynthesis pathway, and intermediates of the riboflavin biosynthesis pathway and their metabolites allows the immune system to rapidly sense pathogen-associated molecular patterns that are shared by a wide range of micro-organisms. Given the essential nature of these metabolic pathways for microbial viability, they have emerged as promising targets for the development of novel antibiotics. Here, we review recent findings that link enzymatic inhibition of microbial metabolism with alterations in the levels of unconventional T cell ligands produced by treated micro-organisms that have given rise to the concept of ‘immuno-antibiotics’: combining direct antimicrobial activity with an immunotherapeutic effect via modulation of unconventional T cell responses.

## Graphical Abstract

**Graphical Abstract:**
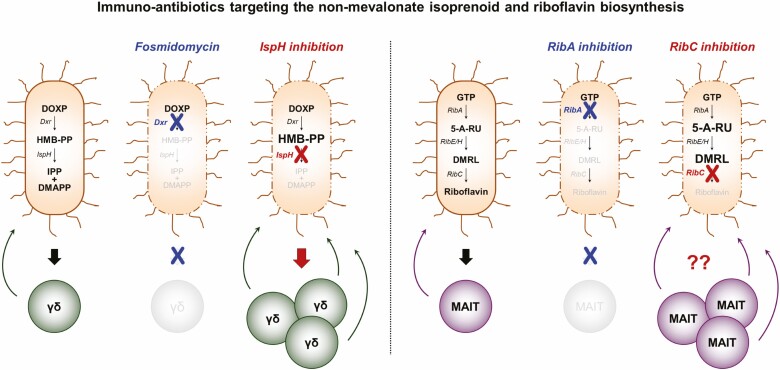

## Introduction

Unconventional T cells represent a class of T cells that—unlike conventional CD4^+^ and CD8^+^ T cells—play a crucial role in sensing danger in the absence of classical restriction via the major histocompatibility complex (MHC). Unconventional T cells comprise both αβ and γδ T cells that survey peripheral tissues and respond to stress-related changes upon infection, injury, and malignancy. In some instances, such responses include the recognition of non-peptidic antigens, typically in the context of members of the CD1 family or the MHC-related protein MR1, or via others factors such as butyrophilins [1]. There are two well-characterised populations of unconventional T cells which stand out with regard to their relative abundance in the human body, their broad reactivity towards microbial metabolites shared by a wide range of pathogens, and their ease of manipulation *in vitro*: Vγ9/Vδ2 T cells (also called Vγ2/Vδ2 T cells according to an alternative nomenclature) and mucosal-associated invariant T (MAIT) cells [2]. Moreover, there is the possibility of a compensatory interplay between these two types of unconventional T cells, as suggested from findings in an immunocompromised patient lacking functional MAIT cells, a result of a rare mutation in MR1, who instead showed highly elevated levels of circulating Vγ9/Vδ2 T cells [3]. In the following, we review progress and prospects for targeting microbial metabolic pathways for both direct as well as indirect, unconventional T cell-based killing of pathogens, as well as immunomodulation in general.

### ‘Phosphoantigen’-reactive γδ T cells

Vγ9/Vδ2 T cells are the dominant population of γδ T cells in human blood where they typically constitute 1–5% of all circulating T cells, but this can increase to >20–40% in many infections [4]. They are characterised by a distinct T cell receptor (TCR) composed of a semi-invariant Vγ9-JP (TRVG9–TRJP) chain and a highly diverse Vδ2 (TRVD2) chain. Intriguingly, Vγ9/Vδ2 T cells have only been found in higher primates and alpacas, being absent in all other animal species studied so far [5]. They respond rapidly to intermediates of the isoprenoid biosynthesis, such as isopentenyl pyrophosphate (IPP) and (*E*)-4-hydroxy-3-methyl-but-2-enyl pyrophosphate (HMB-PP), which bind to the intracellular B30.2 domain of butyrophilin BTN3A1 [6–8]. It is thought that this interaction induces a conformational change of the intracellular part of the protein and that this modified BTN3A1, and the related butyrophilin BTN2A1—possibly together with as yet unknown cellular product(s)—form a complex on the cell surface that triggers the TCR-mediated activation of Vγ9/Vδ2 T cells [5, 9]. As such, IPP and HMB-PP are not TCR ligands themselves but rather facilitate BTN2A1/BTN3A1-dependent γδ T cell responses towards microbial infections, in a truly unconventional fashion compared to ‘classical’ antigens. The precise mechanism of this enigmatic recognition is yet to be unveiled.

### HMB-PP generation by micro-organisms

HMB-PP is a highly immunogenic intermediate of the so-called non-mevalonate (or MEP, after its signature metabolite 2-*C*-methyl-d-erythritol 4-phosphate) pathway of isoprenoid biosynthesis that is utilised by a wide range of Gram-negative and Gram-positive bacteria but not by some bacterial pathogens, such as *Staphylococcus* spp. and *Streptococcus* spp., and yeasts [10]. Of note, the MEP pathway is also present in the chloroplasts of plants and in the plastid-like organelles of apicomplexan parasites, such as *Plasmodium falciparum* and *Toxoplasma gondii*, but is absent in animals.

**Figure 1:**
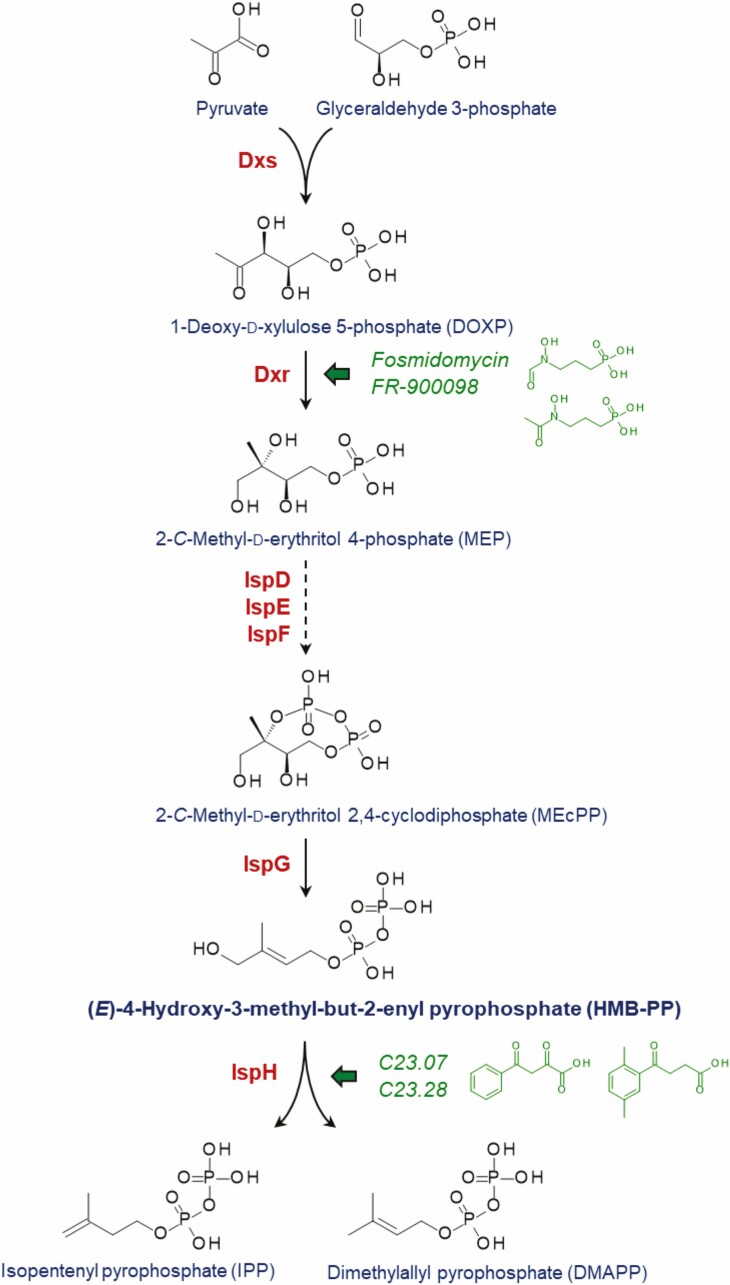
Reaction steps of the MEP pathway of isoprenoid biosynthesis and generation of the Vγ9/Vδ2 T cell activator HMB-PP. Red colour denotes the individual enzymes involved in the pathway; green arrows depict the targets of the Dxr inhibitors fosmidomycin and FR-900098, and the IspH inhibitors 2,4-dioxo-4-phenylbutanoate (C23.07) and 4-(2,5-dimethylphenyl)-4-oxobutanoate (C23.28). Enzymes: Dxs, 1-deoxy-d-xylulose 5-phosphate (DOXP) synthase; Dxr, DOXP reductoisomerase; IspD, 2-*C*-methyl-d-erythritol 4-phosphate (MEP) cytidylyltransferase; IspE, 4-diphosphocytidyl-2-*C*-methyl-d-erythritol kinase; IspF, 2-*C*-methyl-d-erythritol 2,4-cyclodiphosphate (MEcPP) synthase; IspG, (*E*)-4-hydroxy-3-methyl-but-2-enyl pyrophosphate (HMB-PP) synthase (GcpE); IspH, HMB-PP reductase (LytB).

The MEP pathway starts from the condensation of pyruvate and glyceraldehyde 3-phosphate (Fig. 1) and proceeds via the key enzymes 1-deoxy-d-xylulose 5-phosphate (DOXP) reductoisomerase (Dxr), HMB-PP synthase (IspG/GcpE) and HMB-PP reductase (IspH/LytB), yielding a mixture of the end products IPP and dimethylallyl pyrophosphate (DMAPP) in a ~5:1 ratio [10]. IPP and DMAPP are the building blocks of all higher isoprenoids, which include ubiquinone and menaquinone, essential for electron transport and ATP generation, as well as in bacteria long chain isoprenyl pyrophosphates such as undecaprenyl pyrophosphate, essential for cell wall (peptidoglycan) biosynthesis. Since it is absent in humans, the MEP pathway has, therefore, emerged as an attractive drug target for a wide range of infections [11, 12], both bacterial and protozoal.

### The MEP pathway as drug target

Historically, the best characterised target in the MEP pathway is Dxr, with the inhibitor fosmidomycin entering clinical trials against urinary tract infections in the mid 1980s [13], and against malaria, since 2003 [14]. Subsequent studies have also demonstrated the efficacy of fosmidomycin against multidrug-resistant bacteria [15]. Despite only having been discovered relatively recently, the crystal structures of all seven enzymes of the MEP pathway have been solved, often from more than one organism, and the enzymatic reactions they catalyse have been fully elucidated [16]. As a result, inhibitors of each enzyme have been characterised, and some of these compounds have been shown to inhibit bacteria and/or malaria parasites *in vitro* [17–21]. More recently, Singh *et al*. [22] showed the efficacy of novel IspH inhibitors against pan-resistant or multidrug-resistant strains of *Acinetobacter baumannii*, *Enterobacter aerogenes*, *Klebsiella pneumoniae*, *Pseudomonas aeruginosa*, and *Vibrio cholerae in vitro*, and against *Enterobacter aerogenes* in mice, and importantly, these compounds also affect the immune system: ‘immuno-antibiotics’ [23].

### Genetic manipulation of HMB-PP biosynthesis

Given the importance of the MEP pathway for the generation of the γδ T cell activator HMB-PP, any manipulation of the pathway that results in an alteration of HMB-PP production is likely to have an impact on the corresponding Vγ9/Vδ2 T cell response, as summarised in Table 1. For example, targeted deletion of genes encoding enzymes upstream of the generation of HMB-PP leads to a drastically reduced capacity of bacterial mutants to activate Vγ9/Vδ2 T cells [24–27]. In contrast, overexpression of Dxs [28] or IspG [28–30] enhances the metabolic flux through the pathway, thereby increasing HMB-PP levels, as does the targeted deletion of IspH [22, 25, 27, 31, 32], with HMB-PP accumulating and leading to Vγ9/Vδ2 T cell stimulation. This correlation between bacterial HMB-PP levels and the ensuing γδ T cell response is true not only *in vitro* but has also been demonstrated in animal models in which deletion of IspG in *Listeria monocytogenes* reduced γδ T cell responses in rhesus macaques compared to wildtype *Listeria* [26, 33], while deletion of IspH resulted in enhanced γδ T cell responses to *E. coli* in humanised mice [27] and to *Salmonella enterica* in rhesus macaques [32].

**Table 1:** Immunological consequence of manipulating the MEP pathways of isoprenoid biosynthesis

Organism	Target enzyme	Type of manipulation	Effect on γδ T cell response	Experimental model	Ref.
*Plasmodium falciparum*	Dxr	Inhibition	Reduction ↘	*in vitro*	[35, 36]
*E. coli*	Dxr	Genetic deletion	Reduction ↘	*in vitro*	[24, 27]
*E. coli*	Dxr	Inhibition	Reduction ↘	*in vitro*	[30, 34]
*E. coli*	IspG (GcpE)	Genetic deletion	Reduction ↘	*in vitro*	[24, 27]
*E. coli*	IspH (LytB)	Genetic deletion	Enhancement ↗	*in vitro*	[22, 27, 31]
*E. coli*	IspH (LytB)	Inhibition	Enhancement ↗	*in vitro*	[22]
*E. coli*	IspH (LytB)	Genetic deletion	Enhancement ↗	Hu-PBL-SCID/beige mice	[27]
*E. coli*	IspH (LytB)	Inhibition	Enhancement ↗	Hu-PBL-NSG mice	[22]
*Listeria innocua*	IspG (GcpE)	Overexpression	Enhancement ↗	*in vitro*	[29, 30]
*Listeria monocytogenes*	IspG (GcpE)	Genetic deletion	Reduction ↘	*in vitro*	[25, 26]
*Listeria monocytogenes*	IspG (GcpE)	Genetic deletion	Reduction ↘	Rhesus macaques	[26, 33]
*Listeria monocytogenes*	IspH (LytB)	Genetic deletion	Enhancement ↗	*in vitro*	[25]
*Mycobacterium smegmatis*	IspG (GcpE)	Overexpression	Enhancement ↗	*in vitro*	[30]
*Mycobacterium smegmatis*	IspH (LytB)	Inhibition	Enhancement ↗	*in vitro*	[22]
*Mycobacterium tuberculosis*	Dxs	Overexpression	Enhancement ↗	*in vitro*	[28]
*Mycobacterium tuberculosis*	Dxr	Overexpression	No effect —	*in vitro*	[28]
*Mycobacterium tuberculosis*	IspG (GcpE)	Overexpression	Enhancement ↗	*in vitro*	[28]
*Salmonella enterica* ser. Typhimurium	IspH (LytB)	Genetic deletion	Enhancement ↗	*in vitro*	[32]
*Salmonella enterica* ser. Typhimurium	IspH (LytB)	Genetic deletion	Enhancement ↗	Rhesus macaques	[32]
*Vibrio cholera*	IspH (LytB)	Inhibition	Enhancement ↗	*in vitro*	[22]

Hu-PBL-SCID/beige: Mice displaying severe combined immunodeficiency (SCID) affecting both B and T cells and carrying the beige mutation resulting in defective natural killer cells; reconstituted with human peripheral blood lymphocytes.

Hu-PBL-NSG: Non-obese diabetic (NOD), severe combined immunodeficiency (SCID) and IL-2 receptor common gamma deficient mice lacking mature B, T and NK cells; reconstituted with human peripheral blood lymphocytes.

### Modulation of γδ T cell responses by immuno-antibiotics

The findings with genetically engineered bacteria hold true for the pharmacological inhibition of the MEP pathway. For example, inhibition of Dxr using fosmidomycin abrogated Vγ9/Vδ2 T cell responses to *E. coli* [34], *Enterobacter cloacae* [30] and *Plasmodium falciparum in vitro* [35, 36], while inhibition of IspH increased anti-microbial Vγ9/Vδ2 T cell responses to *E. coli*, *Mycobacterium smegmatis* and *Vibrio cholerae in vitro,* and to *E. coli* in humanised mice [22]. Based on these observations, the MEP pathway not only constitutes a promising target for antimicrobial and antimalarial therapy [21, 37, 38], but also allows for the possibility of developing antibiotics that deliberately modulate Vγ9/Vδ2 T cell responses as a second effect (Table 1). Depending on the enzymatic target, this may either result in silencing microbe-responsive Vγ9/Vδ2 T cells—for instance, when using fosmidomycin—or in ‘turbo-charging’ Vγ9/Vδ2 T cells, as in the case of IspH inhibitors.

Which of these two options is most desirable depends on the clinical context. In vulnerable individuals such as patients with chronic kidney disease who depend on peritoneal dialysis as a life-saving renal replacement therapy, any inflammation-related damage to the peritoneal membrane will negatively affect short and long-term clinical outcomes, so the possibility of silencing pro-fibrotic Vγ9/Vδ2 T cell responses during episodes of acute bacterial peritonitis might be an attractive option [39]. Similarly, particular attention needs to be paid to the protection of other organs that are prone to inflammation-related damage, like the lungs, eyes, brain, or reproductive organs. Notwithstanding this potential risk, boosting γδ T cell responses towards microbial pathogens is likely to be beneficial in clearing many infections, as shown in humanised mice where a productive Vγ9/Vδ2 T cell expansion was associated with far lower bacterial loads in different organs [22]. In support of a protective role, adoptive transfer of Vγ9/Vδ2 T cells to *Mycobacterium tuberculosis*-infected cynomolgus macaques led to lower bacterial burdens in the lung and other organs, and attenuated the tuberculosis-associated pathology [40]. Moreover, a modified *Salmonella* vaccine strain harbouring a deletion of IspH, thereby stimulating Vγ9/Vδ2 T cells better than the parental strain, was considered safe with no apparent side effects, in rhesus macaques [32]. It is thinkable that harnessing HMB-PP specific Vγ9/Vδ2 T cells using immuno-antibiotics may in fact boost memory-like recall responses to reinfection by HMB-PP producing organisms, and contribute to cross-protection against unrelated microbial species [41].

### Inhibition of the classical isoprenoid pathway in humans and microbes

IPP is present in all cells and is generated either from HMB-PP via the MEP pathway or, in organisms that do not produce HMB-PP (including humans), via the classical mevalonate pathway [10, 12]. Free IPP is about 10,000 fold less active than is HMB-PP in stimulating Vγ9/Vδ2 T cells in culture [10], and about 1000 fold less active in binding to the purified BTN3A1 protein [6, 8]. Despite this low bioactivity *in vitro*, IPP may still play a physiological role as a Vγ9/Vδ2 T cell activator by indicating the metabolic status of host cells. In fact, pharmacological inhibition of downstream enzymes of the mevalonate pathway that increases intracellular IPP levels readily triggers Vγ9/Vδ2 T cell responses towards treated cells. The most studied examples are with the enzyme farnesyl pyrophosphate synthase (FPPS) that condenses two IPP molecules with one DMAPP to produce the C_15_ species, farnesyl pyrophosphate. Inhibiting the expression of FPPS [42], or blocking its activity using aminobisphosphonates such as pamidronate or zoledronate [43, 44], leads to accumulation of IPP and DMAPP, both of which activate Vγ9/Vδ2 T cells. Aminobisphosphonates are widely used in the clinic to treat excessive bone resorption in patients with osteoporosis and cancer [45], and because of their potential to induce Vγ9/Vδ2 T cell expansion *in vitro* and *in vivo* they are being exploited as novel immunotherapeutics [46, 47]. While these drugs are generally considered safe, it remains unclear how Vγ9/Vδ2 T cells distinguish between metabolically active tissues that may contain elevated IPP levels upon malignant transformation, and healthy tissues with a naturally high throughput via the mevalonate pathway, such as steroid hormone producing glands and the liver, a major cholesterol producer in the body.

Since the mevalonate pathway is also used by bacteria of clinical relevance, such as *Staphylococcus aureus*, and the bacterial enzymes are sufficiently distinct structurally from their mammalian counterparts, inhibitors of the bacterial isoprenoid biosynthesis are of increasing interest as candidates for novel classes of antibiotics targeting isoprenoid virulence factors and cell wall biosynthesis inhibitors [48, 49]. Whether these inhibitors lead to an accumulation of upstream intermediates including IPP in treated bacteria, and hence an increased capacity to stimulate Vγ9/Vδ2 T cells, thus evoking the effect of aminobisphosphonates on human cells, remains to be determined.

### Immunological consequence of manipulating the riboflavin biosynthesis pathway

**Figure 2:**
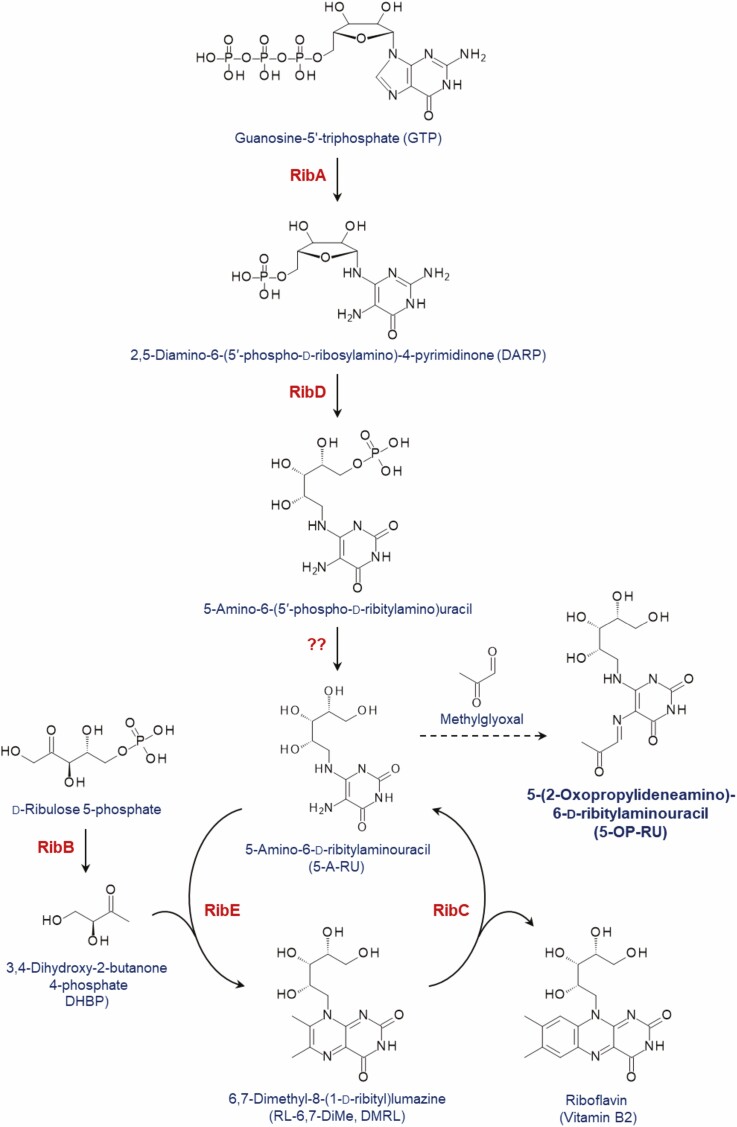
Reaction steps of the riboflavin pathway and generation of the MAIT cell ligand 5-OP-RU. Red colour denotes the individual enzymes involved in the pathway. Enzymes are labelled according to their names in *E. coli*; genes and enzymes in other bacteria such as *Bacillus subtilis* or *Lactococcus lactis* may follow a different nomenclature. Note also that RibC catalyses the dismutation of two DMRL molecules. Enzymes: RibA, guanosine-5’-triphosphate (GTP) cyclohydrolase II; RibB, 3,4-dihydroxy-2-butanone 4-phosphate (DHBP) synthase; RibC, riboflavin synthase; RibD, 2,5-diamino-6-(5′-phospho-d-ribosylamino)-4-pyrimidinone (DARP) deaminase/reductase; RibE, 6,7-dimethyl-8-(1-d-ribityl)lumazine synthase (lumazine synthase);??, hypothetical phosphatase.

Vγ9/Vδ2 T cells are not the only unconventional human T cells that recognise microbial metabolites. Another well-described antimicrobial T cell population are MAIT cells that recognise intermediates of the microbial riboflavin (vitamin B2) biosynthesis pathway presented by the MHC-related molecule MR1 [50]. While the majority of bacteria and fungi possesses the riboflavin biosynthesis pathway, prominent exceptions being *Streptococcus* spp. and *Enterococcus* spp., it is essential and is notably absent in humans and other animals, so constitutes a potential drug target [51–53]. By analogy to the MEP pathway, the riboflavin biosynthesis pathway can be interrupted at various steps that are upstream or downstream of the generation of the potent MAIT cell ligand, 5-(2-oxopropylideneamino)-6-d-ribitylaminouracil (5-OP-RU) [50] (Fig. 2). In fact, genetic deletion of the enzymes mediating early steps in the pathway has been shown to abrogate MAIT cell responses to *Lactococcus lactis*, *E. coli* and *Salmonella typhimurium in vitro* [54–56] and to *E. coli* and *Salmonella typhimurium* in experimental mouse models *in vivo* [55–57], mimicking the effect of Dxr or IspG deficiency on Vγ9/Vδ2 T cells (Table 2). But how to improve activity?

**Table 2:** Immunological consequence of manipulating the riboflavin pathway

Organism	Target enzyme	Type of manipulation	Effect on MAIT cell response	Experimental model	Ref.
*E. coli*	GTP cyclohydrolase II (RibA)	Genetic deletion	Reduction ↘	*in vitro*	[55]*
*E. coli*	GTP cyclohydrolase II (RibA)	Genetic deletion	Reduction ↘	iVa19-Tg mice	[55]*
*E. coli*	3,4-Dihydroxy-2-butanone 4-phosphate synthase (RibB)	Genetic deletion	No effect —	*in vitro*	[55]*
*E. coli*	Pyrimidine deaminase/reductase (RibD)	Genetic deletion	Reduction ↘	*in vitro*	[55]*
*E. coli*	Pyrimidine deaminase/reductase (RibD)	Genetic deletion	Reduction ↘	iVa19-Tg mice	[55]*
*E. coli*	Pyrimidine deaminase/reductase (RibD)	Genetic deletion	Reduction ↘	C57BL/6 mice	[57]*
*E. coli*	Lumazine synthase (RibE)	Genetic deletion	No effect —	*in vitro*	[55]*
*E. coli*	Lumazine synthase (RibE)	Genetic deletion	No effect —	iVa19-Tg mice	[55]*
*E. coli*	*n/a*	Chlorpyrifos treatment	Enhancement ↗	*in vitro*	[63]*
*Lactococcus lactis*	GTP cyclohydrolase II (RibA)	Genetic deletion	Reduction ↘	*in vitro*	[54]
*Lactococcus lactis*	3,4-Dihydroxy-2-butanone 4-phosphate synthase (RibB)	Genetic deletion	No effect —	*in vitro*	[54]
*Lactococcus lactis*	Pyrimidine deaminase/reductase (RibG)	Genetic deletion	Reduction ↘	*in vitro*	[54]
*Lactococcus lactis*	Lumazine synthase (RibH)	Genetic deletion	No effect —	*in vitro*	[54]
*Salmonella typhimurium*	Pyrimidine deaminase/reductase (RibD)and Lumazine synthase (RibH)	Genetic deletion	Reduction ↘	*in vitro*	[54, 56]*
*Salmonella typhimurium*	Pyrimidine deaminase/reductase (RibD)and Lumazine synthase (RibH)	Genetic deletion	Reduction ↘	C57BL/6 mice	[56]*

*Studies were performed using murine MAIT cells, which display the same MR1-dependent reactivity towards riboflavin metabolites as human MAIT cells.

iVa19-Tg: Mice expressing an invariant murine Vα19–Jα33 TCRα chain transgene.

Note that the short names for some of these enzymes (*e.g.* RibE) differ between bacterial species.

The final two steps in the generation of riboflavin are the condensation of 5-amino-6-d-ribitylaminouracil (5-A-RU) and 3,4-dihydroxy-2-butanone 4-phosphate (DHBP) to form 6,7-dimethyl-8-(1-d-ribityl)lumazine (DMRL), catalysed by lumazine synthase (RibE in *E. coli*, RibH in some other bacteria), followed by the dismutation of two DMRL molecules into riboflavin and 5-A-RU, catalysed by riboflavin synthase (RibC). The MAIT cell ligand 5-OP-RU is not an actual intermediate in the riboflavin biosynthesis pathway but rather, is generated when 5-A-RU reacts with methylglyoxal, an endogenous metabolite in mammalian cells and also a constituent in some dietary sources [58]. Evoking the accumulation of HMB-PP in the MEP pathway either by genetic manipulation or via IspH inhibition [22, 31], similar effects in the riboflavin biosynthesis pathway might lead to the accumulation of upstream intermediates including 5-A-RU and DMRL, and to concomitant MAIT cell activation. Indeed, interruption of the terminal step of the pathway can lead to enhanced levels of DMRL in some bacteria, as shown for riboflavin synthase deficient *Bacillus subtilis* [59]. However, things are a little more complicated. Specifically, genetic knockout of GTP cyclohydrolase II (RibA) or pyrimidine deaminase/reductase (RibD/RibG) abrogated 5-OP-RU formation and MAIT cell activation by *E. coli* and *Lactococcus lactis*, as expected, but DHBP synthase (RibB) or lumazine synthase (RibE/RibH) knockouts did not show enhanced 5-OP-RU levels or enhanced MAIT cell activation, compared to wildtype bacteria (Table 2) [54, 55].

A possible reason for these observations is that in order to grow, all of these knockouts require the addition of riboflavin to the medium [54, 55, 59], and high concentrations of riboflavin are known to trigger the so-called flavin mononucleotide (FMN) riboswitch—a genetic element that in many bacteria controls the expression of genes responsible for riboflavin biosynthesis by causing premature termination of the transcription of the rib operon [60, 61]. Thus, a partial shutdown of the pathway via the FMN riboswitch in the presence of exogenous riboflavin would be expected to decrease the anticipated accumulation of intermediates such as 5-A-RU and DMRL, depending on the bacterial species and the culture conditions. FMN riboswitch inhibitors in fact constitute a new class of antibiotics that lead to riboflavin starvation [62], and that are thus also likely to reduce MAIT cell activation. The conclusion is, therefore, that unlike the situation with IspH—if we are to apply the concept of immuno-antibiotics to the riboflavin biosynthesis pathway—then enzymatic inhibition of DHBP synthase (RibB), riboflavin synthase (RibC), and/or lumazine synthase (RibE/RibH), rather than their genetic deletion, will be required. In this respect, it is interesting to note that pre-treatment with the pesticide chlorpyrifos has been shown to enhance the potential of *E. coli* to activate MAIT cells, and that this effect appears to be accompanied by a reduced expression of riboflavin synthase [63].

### Targeting other microbe-responsive unconventional T cells

Further candidates for immuno-antibiotics include inhibitors of mycolic acid biosynthesis in mycobacteria, already an attractive target for novel tuberculosis drugs [64–67], since this is likely to affect the response of CD1b-restricted germline-encoded mycolyl lipid-reactive (GEM) T cells [68]. Similarly, interruption of mycobacterial phosphomycoketide biosynthesis [69, 70] might also affect antimicrobial responses by CD1c-restricted T cells [71]. Another well characterised population of unconventional T cells are CD1d-restricted invariant natural killer T (iNKT) cells, which are activated by glycolipids, especially microbial α-linked glycosphingolipids [72]. Altered iNKT cell activation as a consequence of interference with bacterial metabolism has not yet been described, although this may just be a consequence of our limited knowledge of the details of α-glycosphingolipid biosynthesis [73]. Additional unconventional T cell populations with antimicrobial reactivity exist but are often only poorly characterised.

### Outlook

Taken together, recent progress in our understanding of the MEP isoprenoid and riboflavin biosynthesis pathways has opened up new possibilities in targeting microbial pathogens. This is all the more important in the current era of rapidly growing multidrug resistance to existing treatments, and a worrying shortage of new antibiotics in preclinical and clinical development. Most intriguingly, the link between unconventional T cells and microbial metabolism is now leading to the development of new immunotherapies that deliberately exploit this relationship, by using bespoke ‘immuno-antibiotics’ that target pathogens both directly, by interrupting biosynthesis of vital metabolites, and indirectly, by harnessing the immune system against the infectious agent. However, it will be pivotal to minimise possible side effects such as overshooting responses that may cause local or systemic tissue damage. Of particular interest will be answering the question as to whether specifically activating Vγ9/Vδ2 T cells, or other unconventional T cells, using such ‘immuno-antibiotics’ confers long-lasting protection against reinfection with the same pathogen, or even against other microbes producing the same type of unconventional T cell ligands [33, 41]. Undoubtedly, new opportunities will become ever more apparent as we learn more about the peculiar antimicrobial responsiveness of unconventional human T cell subsets, and their immunopathological context.

## Data Availability

No new data were generated or analysed in support of this research.

## Abbreviations

5-A-RU: 5-amino-6-d-ribitylaminouracil
5-OP-RU: 5-(2-oxopropylideneamino)-6-d-ribitylaminouracil
BTN: butyrophilin
DHBP: 3,4-dihydroxy-2-butanone 4-phosphate
DMAPP: dimethylallyl pyrophosphate
DMRL: 6,7-dimethyl-8-(1-d-ribityl)lumazine
FMN: flavin mononucleotide
FPPS: farnesyl pyrophosphate synthase
GEM T cell: germline-encoded mycolyl lipid-reactive T cell
HMB-PP: (*E*)-4-hydroxy-3-methyl-but-2-enyl pyrophosphate
iNKT cell: invariant natural killer T cell
IPP: isopentenyl pyrophosphate
MAIT cell: mucosal-associated invariant T cell
MEP: 2-*C*-methyl-d-erythritol 4-phosphate
MHC: major histocompatibility complex
MR1: major histocompatibility complex class I-related protein 1
TCR: T cell receptor
